# Being eco-sustainable eaters: the role of chronotype and HEXACO personality traits

**DOI:** 10.3389/fnut.2026.1785503

**Published:** 2026-06-24

**Authors:** Federica Scarpina, Maria Elena Navarra, Sara Mambrini, Ramona De Amicis

**Affiliations:** 1 “Rita Levi Montalcini” Department of Neurosciences, University of Turin, Turin, Italy; 2 Istituto Auxologico Italiano, I.R.C.C.S., Laboratorio Di Ricerche in Neurobiologia Clinica, Ospedale San Giuseppe, Piancavallo (VCO), Italy; 3 IRCCS Istituto Auxologico Italiano, Obesity Unit and Laboratory of Nutrition and Obesity Research, Department of Endocrine and Metabolic Diseases, Milan, Italy; 4 International Center for the Assessment of Nutritional Status and the Development of Dietary Intervention Strategies (ICANS-DIS), Department of Food, Environmental and Nutritional Sciences (DeFENS), University of Milan, Milan, Italy

**Keywords:** chronotype, eating style, ecosustainability, HEXACO, personality

## Abstract

**Background:**

Chronotype and temperament are stable individual traits that influence behaviors such as eating habits. This research examined their impact on an eco-sustainable diet, characterized by a high intake of plant-based foods and reduced consumption of animal products.

**Methods:**

A total of 738 Italian individuals were surveyed regarding their eating habits using a specific food frequency questionnaire. Individual chronotype was assessed via the revised Morningness-Eveningness Questionnaire (rMEQ), while personality traits were measured according to the HEXACO Adjective Scales. The contribution of these factors to the adoption of an eco-sustainable diet was analyzed using hierarchical binary logistic regression.

**Results:**

In our sample, a substantial majority (*N* = 592; 80.22%) reported a non-eco-sustainable diet, with only 146 participants (19.78%) reporting an eco-sustainable diet. Key predictors of an increased likelihood of adhering to an eco-sustainable diet included being female (OR = 3.05, 95% CI [1.85, 5.03]), a greater preference for morningness (OR = 1.08, 95% CI [1.01, 1.14]), higher levels of Agreeableness (OR = 1.43, 95% CI [1.09, 1.86]), and increased Openness to Experience (OR = 1.60, 95% CI [1.23, 2.07]). Conversely, higher scores in Honesty-Humility (OR = 0.69, 95% CI [0.49, 0.97]), Extraversion (OR = 0.80, 95% CI [0.67, 0.96]), and increasing age (OR = 0.98, 95% CI [0.97, 1.00]) were associated with reduced odds.

**Conclusion:**

Both chronobiology and personality traits may significantly influence an individual’s inclination toward eco-sustainable dietary choices. The intrinsic characteristics of Agreeableness and Openness to Experience, but not those associated with Honesty-Humility and Extraversion, may facilitate individuals in addressing the contextual challenges of adopting an eco-sustainable diet.

## 1 Introduction

Chronotype and temperamental traits interact in influencing how individuals engage with and cope with personal needs, including food intake, and environmental and social demands (1) . Interestingly, both are quite stable traits of within-individual functioning. Indeed, chronotype refers to the inter-individual variations in rest/activity patterns or circadian timing. It is conceptualized as a spectrum ranging from extreme morning chronotype (i.e., a predilection for morning hours) to extreme evening chronotype (i.e., a predilection for evening hours), with some individuals exhibiting an intermediate preference (2, 3). Temperamental traits are stable psychological characteristics that influence individuals’ attitudes and behaviors across different contexts (American Psychological Association [APA], (4)). In psychological literature, various theoretical approaches describe temperamental traits, such as the HEXACO model of personality (5): it posits personality to be a set of fixed traits, including Neuroticism, Extraversion, Openness, Agreeableness, and Conscientiousness, as the Big Five Model (6), and Honesty-Humility. As previously stated, chronotype and personality traits exhibit an interactive relationship in influencing an individual’s response to both external and internal stimuli. Lenneis et al. observed that individuals with a higher expression of Extraversion and Openness to Experience tend to exhibit later chronotypes, whereas those with a higher expression of Agreeableness and Conscientiousness are more frequently early chronotypes (7). Stevenson et al. found that the dimensions of Type-D personality (characterized by experience of negative affect and social inhibition) and multidimensional perfectionism were associated with a diurnal preference (8). The interaction between chronotype and personality has been observed to influence dietary choices and eating behaviors (Matthews; Diaz-Morales), leading to distinct eating patterns and food preferences among individuals with different chronotypes (1, 9). In their review, Mazri and colleagues reported that evening chronotypes are more prone to consuming larger meals later in the day (Mazri et al.), potentially increasing caloric intake and disrupting sleep patterns (10). In terms of macro- and micronutrients and food groups, the circadian preference toward eveningness was observed as associated with lower protein and vegetable intake, as well as increased sucrose, sweets, caffeine, and alcohol consumption, and a lower intake of grains and fruits. Conversely, both morning and evening chronotypes consumed approximately the same number of calories, and similar amounts of carbohydrates, fats, cholesterol, fiber, legumes, meat, fish, and dairy products. Personality traits can influence dietary choices and the type of diet, including individual preferences for healthy or unhealthy food. Traits such as impulsivity and sensation-seeking have been linked to unhealthy dietary choices and disordered eating behaviors, particularly among evening chronotypes (11, 12). In the framework of personality, neuroticism was found to be associated with unhealthy diet habits, such as low consumption of fruit and vegetables, and the increased consumption of sugar and saturated fat, while higher expressions of Conscientiousness and higher expression of Openness to Experience were associated with a larger consumption of fruits and vegetables (13–16). Crucially, Bates and colleagues reported that people with higher expression of Openness-to-Experience were more prone to use meat substitutes, although Agreeableness demonstrated a significant positive correlation with insect consumption (17). Crucially, Ardebili and Rickertsen suggested that adopting a sustainable diet was strongly associated with the expression of Openness-to-Experience (18).

Recently, there has been increasing interest in environmentally sustainable eating habits. The rapid deterioration of Earth’s resources, primarily attributable to human activities such as overconsumption and unsustainable food production practices, contributes to climate change. Consequently, it has become imperative for individuals to transition toward an eco-sustainable diet, defined as a dietary pattern characterized by a greater consumption of plant-based foods and whole grains, coupled with a reduced intake of directly or indirectly derived animal products (i.e., *flexitarian diet)* (19) . To facilitate this process of change, researchers and institutions are actively seeking evidence regarding individual factors that may assist citizens in adopting more eco-sustainable lifestyles, including food consumption. However, despite the urgency, the available evidence remains scarce. A recent study highlighted the role of impulsiveness in adopting an eco-sustainable diet, while another underscored the importance of the emotional value attributed to the behavior (i.e., affect), felt obligation, and social norms (20, 21). From a theoretical standpoint, the relationship between stable individual traits and eco-sustainable behaviors can be analyzed through the lens of self-regulation models and the psychology of values (1, 22). Chronotype represents more than a simple temporal preference; it is intrinsically linked to an individual’s capacity for cognitive control and proactive planning, which are essential for navigating dietary shifts (1, 9, 22). Similarly, the HEXACO model offers a robust framework for understanding how specific traits—such as Agreeableness, reflecting empathy and altruism, and Openness to Experience, reflecting cognitive flexibility—serve as primary motivational drivers for shifting away from conventional dietary norms (5, 23). Within this integrated perspective, the adoption of an eco-sustainable diet is conceptualized as the outcome of an interaction between biological predispositions for self-regulation and a value-based system that prioritizes environmental well-being and altruistic concern (13, 18).

Building upon the aforementioned theoretical framework, the present study aims to investigate the specific influence of chronotype and HEXACO personality traits on the adoption of an environmentally sustainable diet in a sample of Italian adults. While previous research has linked these individual traits to general dietary habits, their role in the specific context of eco-sustainability remains underexplored. We formulated the following hypotheses:

- **Hypothesis 1 (H1):** A preference for morningness will be positively associated with the adoption of an eco-sustainable diet. This is grounded in evidence that early chronotypes often demonstrate higher levels of self-regulation and proactive health behaviors, which are necessary for maintaining non-conventional dietary patterns.
- **Hypothesis 2 (H2):** Higher expressions of Openness to Experience and Agreeableness will predict a greater likelihood of following an eco-sustainable diet. We expect that the cognitive flexibility associated with Openness and the altruistic/empathic nature of Agreeableness will facilitate the transition toward plant-based consumption.
- **Hypothesis 3 (H3):** Conversely, higher levels of Extraversion and Honesty-Humility will be associated with a reduced likelihood of adhering to an eco-sustainable diet. We hypothesize that the social nature of Extraversion may lead to higher conformity with meat-centric social norms, while the facet of Honesty-Humility related to conventionality may inhibit the adoption of dietary patterns perceived as non-conformist.

By testing these hypotheses through a hierarchical regression approach, we aim to demonstrate the incremental validity of psychological variables in explaining sustainable eating behaviors beyond basic demographic factors.

## 2 Methods

### 2.1 Study design and participants

This research project obtained ethical approval from the Institutional Review Board (Approval number Prot. N. 0169980). Participants were thoroughly informed regarding the study’s objectives and the assurance of confidentiality for their responses. Furthermore, they were apprised that all collected data would be de-identified and presented exclusively in aggregated form for statistical analysis. Voluntary electronic consent was secured through a click on a designated section of the webpage, which concurrently functioned as a mechanism for bot detection. Participants retained the right to withdraw from the study at any point, without obligation to furnish a reason, by closing their browser. No compensation was provided. The survey was developed using the LimeSurvey platform and disseminated cross-sectionally from November 1, 2022, to February 28, 2025. Distribution channels included social media, electronic mail, and personal contacts facilitated by the authors. Only adults ( ≥ 18 years of age) residing in Italy at the time of the research and reporting to be proficient in the Italian language were considered eligible for this research. Participants were asked to report gender, age, and education. They also reported weight in kilograms and height in meters: these parameters were used to compute the body mass index according to the following formula: BMI = weight (kg)/[height (m)]2. Given that data were collected through an online survey, specific precautions were taken to minimize and detect potential bot contamination. Participation required active electronic informed consent, which also served as an initial screening step. Data were further screened for implausible response patterns, internal inconsistencies, duplicate entries, and abnormal completion times. No cases meeting criteria suggestive of non-human responding were identified in the final dataset.

Food frequency questionnaire. To assess the eco-sustainability of individual eating habits, we employed a food frequency questionnaire developed by our research group (20). It should be noted that, although the formal publication and validation of this instrument occurred in 2024, the questionnaire was fully developed and implemented for the current study at the onset of data collection in November 2022. Participants rated their weekly consumption of the following food items using a 5-point Likert scale (never, less than once a week, once/twice a week, three or more times a week, daily): whole grains, tubers/starchy vegetables, vegetables, fruits, milk, soft cheese, seasoned cheese, plant-based alternatives to cow’s milk, red meat, processed meat, poultry, eggs, fish, seafood, legumes, oilseed, and tree nuts. An eco-sustainable diet was defined by the following consumption patterns: milk, seasoned and fresh cheese, red and white (including processed) meat, eggs, fish, and seafood consumed never, less than once a week, or once/twice a week; and legumes, whole grains, vegetables, seeds, and nuts consumed three or more times a week, or daily. Notably, vegetarian and vegan diets were classified as eco-sustainable. Participants whose food frequency patterns deviated from these criteria were categorized as having a non-eco-sustainable diet.

#### 2.1.1 Chronotype preference

Chronotype was assessed using a shortened 5-item version of the 19-item Morningness-Eveningness Questionnaire (MEQ) (24). This short version, known as the rMEQ, accounts for 83% of the total variance of the original MEQ, maintaining high reliability and validity, and making it a popular tool for measuring chronotype across various cultures (25) . It captures essential aspects of chronotype, including sleep-wake patterns, peak performance times, and self-perceived chronotype. The rMEQ score ranges from < 12 (extreme evening type) to > 17 (extreme morning type), with intermediate scores representing neutral chronotypes (12–17 points).

#### 2.1.2 HEXACO adjective scales

This tool measures an individual’s expression of the six main personality dimensions of Honesty–Humility, Emotionality, eXtraversion, Agreeableness (vs. anger), Conscientiousness, and Openness to Experience (26). Individuals indicate how each of the 60 Italian adjectives described themselves on a scale from 1 (*“it does not describe me at all”*) to 7 (*“it describes me completely”*). Overall, a higher score suggests a higher expression of the personality trait. The full list of Italian adjectives from the HEXACO Adjective Scales, along with their English translations, the scoring method, and details about the good psychometric properties in terms of internal consistency, convergent validity, nomological validity, criterion validity, and stability of the measure over time, is reported in Romano and colleagues.

### 2.2 Statistical analyses

Data were initially analysed using descriptive statistics, including means, standard deviations, frequencies, and percentages. Scores about the questionnaires were computed according to the normative articles.

Regarding the rMEQ, used to measure chronotype preference, the total score was used as both a continuous variable and a categorical variable. Participants were categorized into tertiles: the lowest tertile represented eveningness, the middle tertile represented intermediate preference, and the highest tertile represented morningness.

To investigate whether personality traits and chronotype predicted the adoption of an eco-sustainable diet above and beyond the influence of demographic factors, a two-step hierarchical binary logistic regression was performed. The dependent variable was the dichotomous classification of eating habits (1 = eco-sustainable diet, 0 = not eco-sustainable diet). In the first block, the demographic and health variables of age (continuous), gender (categorical), and BMI (continuous) were entered as covariates. In the second block, the six continuous scores from the HEXACO Adjective Scales and the continuous total score of the rMEQ were added as the primary predictors. This hierarchical approach allows for testing the incremental validity of the psychological variables after accounting for the baseline variables. For the analysis, we evaluated the overall model fit using the Omnibus Test of Model Coefficients and the Hosmer-Lemeshow test. The explanatory power was assessed using the Nagelkerke R^2^ statistic. The significance of the improvement in model fit between Block 1 and Block 2 was determined by the change in the χ^2^ value. For individual predictors in the final model, Wald statistics were used to assess their significance, and Odds Ratios (OR) with 95% confidence intervals were calculated to interpret the magnitude and direction of the effects.

#### 2.2.1 Sample size

For regression analysis, Green (27) recommends a sample size of at least 50 + 8 m for testing individual coefficients and 104 + m for testing the overall R^2^, where *m* is the number of predictors included in the regression model. Applying these criteria with *m* = 10, the required sample size is 130 participants.

## 3 Results

A total of 738 individuals completed the survey and were included in the sample. The majority of our sample (*N* = 592; 80.22%) reported a non-eco-sustainable diet, while 146 participants (19.78%) reported an eco-sustainable diet [χ^2^(1, *N* = 738) = 269.53, *p* < 0.001]. This significant imbalance aligns with 2024 Italian data from ISTAT (Istituto Nazionale di Statistica) regarding eating habits, which reported that 85.5% of the Italian sample surveyed identified as having an omnivorous diet. Demographic information is reported in Table 1.

**TABLE 1:** Descriptive characteristics of the study sample according to dietary pattern.

Variable	Overall (*N* = 738)	Not eco-sustainable (*N* = 592)	Eco-sustainable (*N* = 146)	*p*-value
Gender (%)				**<0.001**
Male	37.94	41.05	27.40	
Female	61.65	58.95	70.55	
Non-binary	0.41	0.00	2.05	
Age (years), M (SD)	38.18 (15.88)	39.80 (16.32)	35.30 (13.64)	**0.007**
Education (years), M (SD)	15.07 (2.72)	14.94 (2.78)	15.56 (2.40)	**0.007**
BMI (kg/m^2^), M (SD)	23.48 (4.16)	23.70 (4.53)	22.50 (3.18)	**<0.001**
Chronotype Score, M (SD)	15.38 (3.49)	15.28 (3.46)	15.78 (3.58)	0.130
HEXACO Traits, M (SD)				
H	5.95 (0.75)	5.96 (0.78)	5.93 (0.67)	0.660
E	4.29 (0.90)	4.26 (0.92)	4.39 (0.82)	0.120
X	4.62 (1.16)	4.67 (1.77)	4.43 (1.11)	**0.029**
A	5.28 (0.91)	5.25 (0.91)	5.43 (0.90)	**0.036**
C	5.22 (0.97)	5.22 (0.97)	5.20 (0.96)	0.780
O	4.82 (0.85)	4.77 (0.84)	5.01 (0.83)	**0.002**

Significant results (*p* < 0.05) are indicated in bold. M, mean; SD, standard deviation; BMI, body mass index; H, Honesty-Humility; E, Emotionality; X, eXtraversion; A, Agreeableness; C, Conscientiousness; O, Openness to Experience. Statistical differences between groups were assessed using Independent Samples *t*-tests for continuous variables and Chi-square (χ^2^) tests for categorical variables.

The hierarchical binary logistic regression analysis was conducted to predict the likelihood of adopting an eco-sustainable diet. The baseline model containing only demographic variables (age in years, gender, and body mass index) was statistically significant [χ^2^(3) = 23.98, *p* < 0.001], explaining 5.2% of the variance (Nagelkerke R^2^). The addition of the psychological predictors (scores at the HAS scale for the psychological traits and the score at the rMEQ for the chronotype preference) in the second block significantly improved the model fit [Δχ^2^(7) = 34.12, *p* < 0.001]. The final model was statistically significant [χ^2^(10) = 58.09, *p* < 0.001], and explained a total of 12.4% of the variance (Nagelkerke R^2^) in dietary habits. The model demonstrated a good fit to the data, as indicated by a non-significant Hosmer and Lemeshow test [χ^2^(8) = 6.82, *p* = 0.556].

As detailed in Table 2, seven predictors emerged as statistically significant in the final model. Gender was a strong predictor, with being female being over three times more likely to adopt an eco-sustainable diet (OR = 3.05, 95% CI [1.847, 5.029], *p* < 0.001). Increasing age was associated with slightly lower odds of having an eco-sustainable diet (OR = 0.98, 95% CI [0.969, 0.996], *p* = 0.01). Among the psychological variables, higher scores on Agreeableness (OR = 1.43, 95% CI [1.089, 1.864], *p* = 0.01), Openness to Experience (OR = 1.60, 95% CI [1.228, 2.073], *p* < 0.001), and a preference for morningness (higher chronotype score; OR = 1.08, 95% CI [1.014, 1.142], *p* = 0.02) were all associated with increased odds of adhering to an eco-sustainable diet. Conversely, higher scores on Honesty-Humility (OR = 0.69, 95% CI [0.485, 0.971], *p* = 0.03) and eXtraversion (OR = 0.80, 95% CI [0.668, 0.963], *p* = 0.02) were associated with decreased odds of having an eco-sustainable diet. BMI, Emotionality, and Conscientiousness were not significant predictors.

**TABLE 2:** Hierarchical binary logistic regression predicting the likelihood of adopting an eco-sustainable diet.

Predictors	B	S.E.	Wald	*p*	OR [95% CI]
Block 1					
Age	−0.021	0.008	5.720	**0.020**	0.991 [0.973, 0.997]
Gender	0.854	0.220	15.160	**<0.001**	2.350 [1.528, 3.608]
BMI	0.002	0.003	0.950	0.330	1.002 [0.998, 1.006]
Block 2					
Age	−0.022	0.008	6.082	**0.014**	0.978 [0.969, 0.996]
Gender	1.114	0.256	19.030	**<0.001**	3.047 [1.847, 5.029]
BMI	0.003	0.003	1.290	0.260	1.003 [0.998, 1.008]
H	−0.375	0.176	4.531	**0.033**	0.687 [0.485, 0.971]
E	−0.06	0.13	0.25	0.62	0.94 [0.727–1.209]
X	−0.222	0.094	5.571	**0.018**	0.801 [0.668, 0.963]
A	0.354	0.137	6.662	**0.010**	1.425 [1.089, 1.864]
C	−0.18	0.12	2.18	0.14	0.84 [0.666–1.059]
O	0.473	0.135	12.210	**<0.001**	1.605 [1.228, 2.073]
Chronotype Score	0.075	0.031	5.900	**0.015**	1.078 [1.014, 1.142]

Significant results (*p* < 0.05) are indicated in bold. B, Unstandardized coefficient; S.E., Standard Error; Wald, Wald statistic; OR, Odds Ratio; CI, confidence interval. The dependent variable is the dichotomous classification of eating habits (1 = eco-sustainable diet, 0 = not eco-sustainable diet). Block 1 controlled for demographic and health variables (age, gender being female, BMI); Block 2 included the six HEXACO personality dimensions (H, Honesty-Humility; E, Emotionality; X, eXtraversion; A, Agreeableness; C, Conscientiousness; O, Openness to Experience) and the rMEQ chronotype score.

## 4 Discussion

This study sought to provide a preliminary exploration into whether chronotype and personality traits could predict the adoption of an eco-sustainable diet in a sample of Italian adults. Our findings confirm that individual differences in both circadian preference and core personality traits are significantly associated with the likelihood of adhering to a diet characterized by a higher consumption of plant-based foods and a lower intake of animal-origin products. Our results not only corroborate our initial hypotheses about the role of both these psychological components in adopting an ecosustainable diet, but also reveal a more complex and nuanced psychological profile of individuals who choose to have sustainable eating habits. Indeed, according to our results, individuals with a morningness chronotype and with higher expressions of Agreeableness and Openness to Experience may be more prone to adopt an eco-sustainable diet, whereas higher expressions of Honesty-Humility and eXtraversion were linked with a lower likelihood of adopting an eco-sustainable diet.

### 4.1 Theoretical implications

From a theoretical standpoint, the relationship between stable individual traits and eco-sustainable behaviors can be analyzed through the lens of self-regulation models and the psychology of values . Our results suggest that individuals with a morningness chronotype and with higher expressions of Agreeableness and Openness to Experience may be more prone to adopt an eco-sustainable diet, whereas higher expressions of Honesty-Humility and eXtraversion were linked with a lower likelihood of adopting an eco-sustainable diet.

The positive association between morningness and sustainable eating patterns aligns with the “Self-Regulation Hypothesis,” which posits that early chronotypes possess a greater capacity for cognitive control and proactive planning . The adoption and maintenance of an eco-sustainable diet often requires planning, discipline, and a departure from convenience, all of which are characteristics frequently observed in morning-oriented individuals (1, 22, 28).

Similarly, the robust association with Agreeableness, and particularly with Openness to Experience, is conceptually coherent. These results reinforce the role of cognitive flexibility and empathy in dietary transitions. Higher expressions of Openness to Experience characterize individuals who are intellectually inquisitive, creative, and receptive to novelty. Thus, they would be significantly more inclined to embrace non-traditional and sustainable eating patterns. Adopting an eco-sustainable diet frequently requires a willingness to experiment with new foods and recipes, as well as the cognitive flexibility to challenge ingrained cultural and familial dietary norms.

Furthermore, higher expressions of Agreeableness describe individuals demonstrating greater empathy and compassion (29) traits that may foster a heightened concern for environmental sustainability and animal welfare, which are often cited as primary motivators for reducing meat consumption (30–32). The altruistic tendency of Agreeableness acts as a primary motivational driver, supporting individuals to take into account the positive impact of changes toward eco-sustainability on others and future generations (29). This represent a necessary dimension within the context of eco-sustainability, an area still characterized by limited expression and social stigma.

Crucially, our study provides a novel theoretical contribution regarding the negative relationship with eXtraversion and Honesty-Humility. The negative relationship with eXtraversion might be explained by the highly social nature of this trait (29). Extraverted individuals may engage more frequently in social eating occasions where traditional, meat-centric food options are often more prevalent and socially normative. Regarding Honesty-Humility, we propose that the conventionality facet may inhibit the adoption of diets perceived as non-conformist. While this trait typically encompasses sincerity and fairness (29), higher expressions represent a tendency to cooperate without necessarily embracing the altruistic tendency linked to Agreeableness (23). Adherence to conventional norms and values may conflict with the adoption of a dietary pattern which may still be perceived as alternative [Tucker (33); Leone et al., (34)].

According to our results, gender seems to play a role in the eco-sustainable diet, consistent with research showing that women are more likely than men to adopt plant-based diets (35–37). The slight negative association with age suggests that younger cohorts may be more inclined to adopt these patterns, possibly due to greater exposure to information about climate change and sustainability (38). Finally, body mass index was not implicated in the tendency toward eco-sustainability (39–41).

### 4.2 Practical implications

These insights have significant practical applications for public health and environmental policy. Public health campaigns aimed at promoting sustainable diets could be substantially enhanced by tailoring messages to resonate with diverse psychological profiles:

- Considering the evidence about the Openness to Experience trait: messages should emphasize culinary novelty, diversity, and the “adventure” of plant-based cooking to appeal to their receptive nature.
- Since the evidence about the Agreeableness trait: Campaigns should focus on the altruistic impact of dietary choices on animal welfare and future generations.
- Because of the evidence about eXtraversion: Interventions should address social barriers by promoting eco-sustainable options in social eating contexts.

Furthermore, the evidence about the morningness chronotype suggested the importance to provide easy-to-follow meal planning tools to compensate for potential self-regulation challenges.

### 4.3 Limitations and future research

The present study has several strengths, including its novel research question and the use of a robust hierarchical analysis that controlled for key covariates. By demonstrating the incremental validity of psychological traits, we provide a more rigorous foundation for our conclusions. However, some limitations must be acknowledged. First, the cross-sectional design precludes any causal inferences; it is unclear whether personality and chronotype influence ecosustainable diet or if long-term dietary choices can shape aspects of one’s lifestyle that are correlated with these two psychological traits. Second, we used self-report measures, which may be subject to social desirability bias. Third, the sample was collected through online convenience methods, which may not be fully representative of the broader Italian population. Finally, a significant consideration regarding our methodology concerns the operationalization of an ‘eco-sustainable diet.’ In the present study, we defined sustainability primarily through the lens of a plant-forward or ‘flexitarian’ dietary pattern, characterized by high consumption of legumes, whole grains, and vegetables, and low intake of animal-derived products. This approach aligns with the core recommendations of the EAT-Lancet Commission, which emphasizes that reducing animal product consumption is a primary lever for staying within planetary boundaries. However, we acknowledge that this represents a simplified, binary classification of a highly complex and multidimensional construct. A truly sustainable diet also encompasses critical factors not captured in our food frequency questionnaire, such as the reduction of food waste, the prioritization of seasonal and locally sourced products (to minimize “food miles”), and the choice of production methods with lower environmental impacts, such as organic or regenerative farming. While our findings provide valuable insights into the psychological drivers of plant-based transitions, they should be interpreted specifically within the context of food-group choices rather than a comprehensive assessment of total environmental footprint. Future research should aim to incorporate these additional dimensions to offer a more holistic understanding of how personality and chronotype influence the broad spectrum of sustainable eating behaviors. The use of more objective measures, such as dietary records or biomarkers, could validate the self-reported data. Furthermore, future studies could explore the underlying intentions and motivations that mediate the relationship between personality and chronotype in ecosustainable and not non-ecosustainable eating habits. For example, are agreeable individuals primarily driven by empathy for animals, while openness-to-experience individuals are motivated by novelty and health benefits? Investigating these mediating mechanisms would provide deeper insights for crafting effective interventions.

## 5 Conclusion

This study provides compelling evidence that the adoption of an eco-sustainable diet is linked to a complex interplay of stable individual traits, including personality and chronobiology. Our findings suggest that individuals who choose a sustainable diet are not only more likely to be agreeable and exhibit morning chronotype but also may demonstrate high openness to new experiences. These characteristics may better support such individuals to navigate the challenges of eco-sustainability within consumerist and predominantly omnivorous social contexts. Conversely, individuals reporting higher expressions of Honesty-Humility, due to inherent difficulties in deviating from socially accepted norms, and Extraversion, due to increased susceptibility to social involvement in non-eco-sustainable eating contexts, may be less inclined to adopt an eco-sustainable diet. These insights have significant practical implications, suggesting that public health campaigns aimed at promoting sustainable diets could be substantially enhanced by tailoring messages to resonate with diverse psychological profiles. By understanding the individual behind the food choices, we can better support the collective shift toward a healthier and more sustainable future.

## Data Availability

The datasets presented in this study can be found in online repositories. The names of the repository/repositories and accession number(s) can be found below: https://doi.org/10.5281/zenodo.17348175.
